# Orthopoxvirus DNA in Eurasian Lynx, Sweden

**DOI:** 10.3201/eid1704.091899

**Published:** 2011-04

**Authors:** Morten Tryland, Malachy Ifeanyi Okeke, Carl Hård af Segerstad, Torsten Mörner, Terje Traavik, Marie-Pierre Ryser-Degiorgis

**Affiliations:** Author affiliations: Norwegian School of Veterinary Science, Tromsø, Norway (M. Tryland);; University of Tromsø, Tromsø (M.I. Okeke, T. Traavik);; National Veterinary Institute, Uppsala, Sweden (C. Hård af Segerstad, T. Mörner, M.-P. Ryser-Degiorgis);; Genøk—Centre for Biosafety, Tromsø (M.I. Okeke, T. Traavik);; University of Bern, Bern, Switzerland (M.-P. Ryser-Degiorgis)

**Keywords:** Cowpox virus, epidemiology, orthopoxvirus, wildlife, zoonosis, viruses, lynx, research

## Abstract

Cowpox virus, which has been used to protect humans against smallpox but may cause severe disease in immunocompromised persons, has reemerged in humans, domestic cats, and other animal species in Europe. Orthopoxvirus (OPV) DNA was detected in tissues (lung, kidney, spleen) in 24 (9%) of 263 free-ranging Eurasian lynx (*Lynx lynx*) from Sweden. Thymidine kinase gene amplicon sequences (339 bp) from 21 lynx were all identical to those from cowpox virus isolated from a person in Norway and phylogenetically closer to monkeypox virus than to vaccinia virus and isolates from 2 persons with cowpox virus in Sweden. Prevalence was higher among animals from regions with dense, rather than rural, human populations. Lynx are probably exposed to OPV through predation on small mammal reservoir species. We conclude that OPV is widely distributed in Sweden and may represent a threat to humans. Further studies are needed to verify whether this lynx OPV is cowpox virus.

Cowpox virus (family *Poxviridae*, genus *Orthopoxvirus* [OPV]) was originally considered to infect milking cows and to have zoonotic potential. Because of its relationship to variola virus and immunologic cross-reaction, cowpox virus was used to protect humans against smallpox (*1*). Later, vaccinia virus, another OPV with unknown origin, was used as a vaccine virus through the global smallpox eradication campaign, and cowpox virus infections became less common in cattle, other animals, and humans. However, during recent decades, cowpox virus infections have reemerged in domestic cats and other animals, including wild animals in captivity (*2**–**4*), and have increased in humans subsequent to transmission from cats, rodents, zoo animals, and circus animals (*5**–**9*). Cowpox virus seems to be restricted to Eurasia, but spread of poxviruses to new regions through relocation of their natural host species is possible (*10*).

Cowpox virus was first isolated from felid species—lions (*Panthera leo*), cheetahs (*Acinonyx jubatus*), black panthers (*Panthera* *pardus*), ocelots (*Leopardus pardalis*), jaguars (*Panthera onca*), pumas (*Felis concolor*), leopard cat (*Prionailurus bengalensis*), Pallas cat (*Otocolobus manul*), and domestic cat (*Felis catus*)—during an outbreak in the Moscow Zoo in 1973–1974 (*11*) and subsequently from 3 cheetahs in Whipsnade Park, England, in 1977 (*12*). Since 1978, cowpox virus infections have been diagnosed in domestic cats in several European countries (*13**–**16*); the cats were probably infected by contact with rodents, and in some cases infections were transmitted to humans (*16**–**18*). Serologic surveys and detection of OPV DNA by PCR have indicated that wild rodents and shrews are likely reservoir hosts of cowpox virus in western Europe (*19**–**23*), although infectious virus has never been isolated from these animals.

Anti-OPV antibodies have been demonstrated in carnivores such as European lynx (*Lynx lynx*), red fox (*Vulpes vulpes*), and a brown bear (*Ursus arctos*) (*24**,**25*). Anti-OPV seroprevalence was 29% among 17 lynx from the Sarek National Park, northern Sweden, and 1% among 73 lynx from southern Finland (*25*). Taking into account the human cowpox virus cases reported from Finland, Sweden, and Norway (*16**,**23**,**26*), OPV, presumably cowpox virus, is probably widespread among wildlife in Scandinavia, having small wild rodent populations as a reservoir. Because carnivores are exposed to OPV through predation on rodents, virus-specific DNA or antibodies in carnivores could serve as an indicator for the epizootiologic situation in rodent populations (*25*).

To our knowledge, no cowpox virus case has been reported in free-ranging wild felids. To evaluate the possibility that cowpox virus may cause disease among Eurasian lynx, we searched for evidence of OPV infection in this large, free-ranging felid in Sweden. We used PCR to detect OPV DNA in tissue samples and compared these data with pathological findings. In addition, to address the possible effects of such viruses in the ecosystems, we searched for phylogenetic relationships between the virus infecting lynx and viruses causing clinical cowpox cases in Scandinavia.

## Materials and Methods

### Animals

Lynx samples were collected as part of a large study assessing the health of the free-ranging lynx population in Sweden from 1989 through 1999 and providing baseline data on diseases and parasites of the Eurasian lynx. Complete necropsy was performed on all animals shipped to the National Veterinary Institute, Uppsala, Sweden, during the study period. Samples of lung, kidney, and spleen were collected during necropsy and stored at –20°C until analysis. The main causes of death were traffic accidents and sarcoptic mange (*Sarcoptes scabiei* infestation) (*27*). Infections with common feline viruses were rare (*28*), and seropositivity to *Toxoplasma gondii* was more common among lynx from central Sweden than from farther north, where the climate is more harsh and the human population less dense (*29*). A total of 263 lynx, sampled during 1995–1999, were included in the present study.

**Figure 1:**
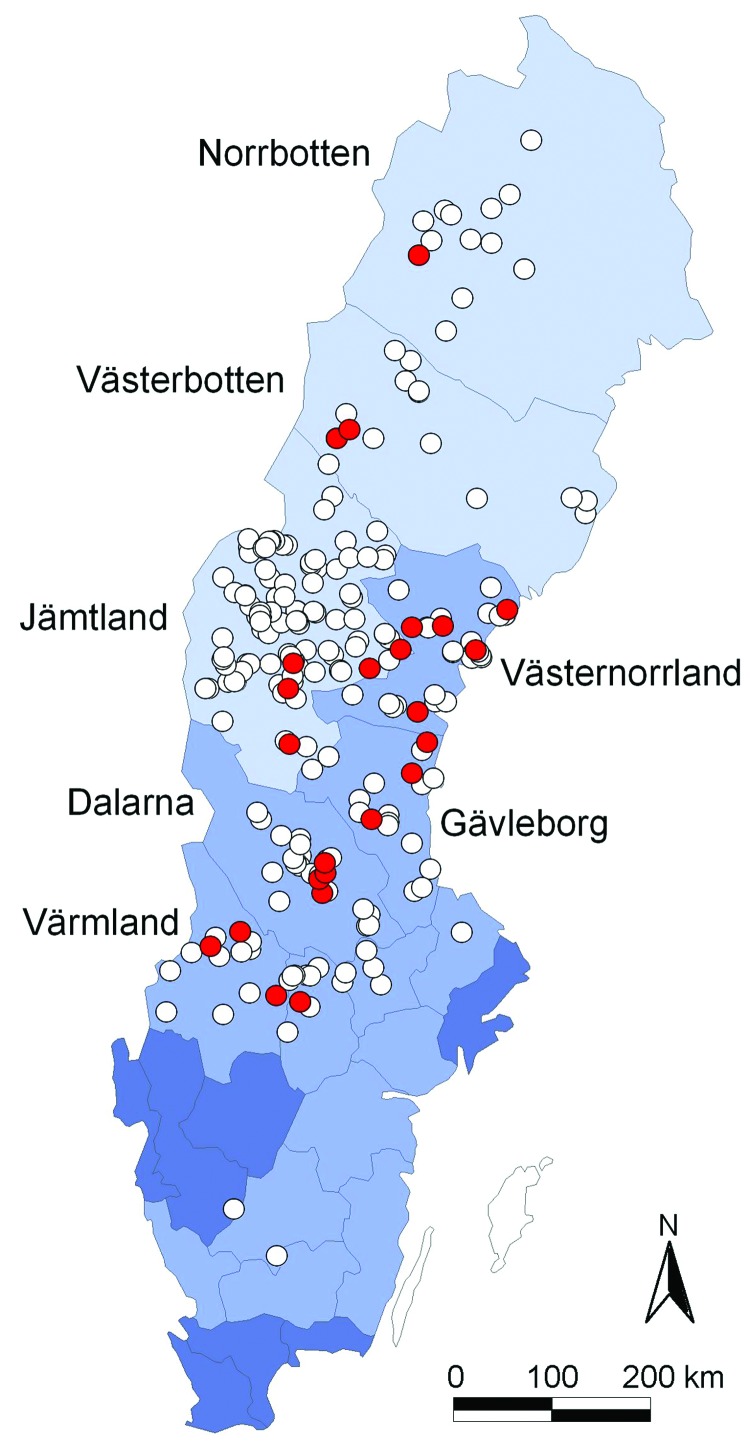
Geographic origin of 263 Eurasian lynx (*Lynx lynx*) collected in Sweden during 1995–1999 and tested for orthopoxvirus (OPV)–specific DNA (open circles). OPV DNA was amplified by PCR from 24 animals (9%; red circles). Light blue areas represent sparsely populated (<5 inhabitants/km^2^) mountainous counties; medium blue areas represent more densely populated counties (10–41 inhabitants/km^2^) farther south; and dark blue areas represent counties with the highest human population densities (>50 inhabitants/km^2^).

Lynx originated from all over Sweden. We differentiated 3 main geographic regions according to human population density (Figure 1). Northern Sweden, apart from the mountain chain bordering Norway, is part of the circumpolar northern coniferous forest belt, the taiga. It is sparsely populated by humans; human populations increase toward the south and gradually decline farther from the coast. The southernmost part of Sweden belongs to the broad-leaf forest region of central Europe but is mainly cultivated. Lynx typically live in forested areas and are found throughout most of Scandinavia. They may come close to human settlements. Although their main prey are roe deer, they also prey on reindeer and sheep. Furthermore, debilitated lynx affected by sarcoptic mange and orphans searching for easily accessible food are often found around human settlements. Interactions between diseased lynx and domestic cats and dogs have been documented (*30**,**31*).

Most (245) animals submitted were apparently healthy (225 killed by hunters, 19 died of traumatic injury, 1 died of acute circulatory failure during anesthesia), 15 had sarcoptic mange, and 3 died of starvation caused by noninfectious or unclear etiology. Lynx killed by hunters were submitted for necropsy without their fur. Body condition was evaluated by body weight, fat deposits in the abdomen and on the heart, and appearance of the bone marrow of the femur. The animals were categorized into 3 groups: normal body condition (normal to fat, n = 230), poor body condition (low amount of fat in abdomen and on heart, n *=* 15), and emaciated (no abdominal fat, serous atrophy of fat deposits on heart and of bone marrow, n *=* 18, i.e., all diseased animals).

For 228 animals, age was determined by counting cementum annuli of a canine tooth (Matson’s Laboratory, Milltown, MT, USA); for the others, it was estimated on the basis of body size and weight, tooth wear, development of genital organs, and, if appropriate, morphologic characteristics and ossification of the skull. Investigated lynx with exact age known were 0–13 years of age (mean 2.5 years ± 2.6 SD). On the basis of sexual maturity, and thus reflecting social behavior, 3 age classes were established: juvenile (<1 year; n *=* 64), subadult (2 years for females and 2–3 years for males; n *=* 95), and adult (>2 years for females and >3 years for males; n *=* 104). Numbers of males and females were equal in the juvenile and adult age classes, but within subadults there were more males (n *=* 68) than females (n *=* 27). According to the time of death, animals were grouped into seasons relevant to the biology of the lynx: delivery (May–July, n *=* 6); small kittens and lactation (August–October; n *=* 7); large kittens hunting with the dam (November–January, n *=* 24); and breeding season, separation of kittens from dam, and main hunting season (February–April, n *=* 224).

### PCR, Electrophoresis, and Sequencing

From each sampled lynx, 25 mg lung (n *=* 262), 25 mg kidney (n *=* 263), and 10 mg spleen (n *=* 261) were cut in small pieces and homogenized in phosphate-buffered saline. DNA was extracted by using a QIAamp tissue kit (QIAGEN GmbH, Düsseldorf, Germany); mean DNA concentration was 39.7 μg/mL ± 23.8 SD for lung, 99.0 μg/mL ± 46.4 SD for kidney, and 101.0 μg/mL ± 49.0 SD for spleen. Five μL of the DNA eluate was used as template for the PCR.

PCR primers (MedProbe AS, Oslo, Norway) from the thymidine kinase gene (*tk*) were used as described (*21*), generating an expected PCR amplicon of 339 bp. DNA from vaccinia virus (Western Reserve; VR-119), cowpox virus (Brighton; VR-302) (both from American Type Culture Collection, Rockville, MD, USA), and cowpox virus isolated from a felid with clinical disease (*16*) was used as positive control. PCR was performed with a Gene Amp PCR System 9700 (PerkinElmer Corp., Norwalk, CT, USA). Reaction volume was 50 μL and contained 5 μL of the DNA eluate from the lynx tissue, 1 μL of each primer (25 μM), 4 μL dNTP (10 mmol/L), 5 μL MgCl_2_ (25 mmol/L), 5 μL GeneAmp 10X Gold Buffer (Applied Biosystems, Oslo, Norway), 2.5 U AmpliTaq Gold polymerase (Applied Biosystems), and 19 μL water. The tubes were placed in a preheated block (95°C) and held for 5 min before cycling. Five cycles of denaturation (95°C for 30 s), annealing (53°C for 2 min), and primer extension (72°C for 30 s) were followed by 35 cycles with 30 s annealing time. After the last cycle, the temperature was held at 72°C for 10 min.

PCR amplicons were analyzed by using the Gibco BRL Horizon 11-14 Gel Electrophoresis System (Life Technologies, Paisly, Scotland) in a 2% agarose gel (UltraPure agarose gel; Life Technologies) with TAE buffer (0.04 M Tris-acetate, 1.0 mmol/L EDTA) and ethidium bromide for DNA staining. Fifteen μL of PCR product mixed with 3 μL 6X loading buffer (0.25% [wt/vol] bromphenol blue and 40% [wt/vol] sucrose) were loaded in each well. The gels were run in TAE buffer at 150 V for 1.5 h and examined by using a Gel Doc 2000 Documentation System (Bio-Rad Laboratories, Oslo, Norway). Primers and dNTP were removed from amplicons by using ExoSapIT reagent (Amersham Pharmacia, Uppsala, Sweden), adding 1 µL/5µL PCR product, incubating 45 min at 37ºC, and by conducting enzyme inactivation for 20 min at 80ºC. Cycle sequencing was conducted in both directions by using Big Dye 3.1 reagents (ABI BigDye Terminator version 3.1, Applied Biosystems). Two microliters EDTA (125 mmol/L), 2 μL sodium acetate (3 mol/L), and 50 μL of ethanol were added to the 20 μL sequencing product. Electrophoresis of the cycle sequencing extension products was conducted in an ABI Prism 377 DNA Analyzer (Applied Biosystems). Raw sequence data were edited by Chromas Pro software version 1.41 (Technelysium Pty Ltd., Tewantin, Queensland, Australia) and BioEdit Sequence Alignment Editor version 7.0.4 (www.mbio.ncsu.edu/BioEdit/bioedit.html). Multiple sequence alignment was performed by using ClustalX 1.181 (*32*). The phylogenetic tree was generated with the neighbor-joining method by using MEGA 3.1 (www.megasoftware.net). The reliability of the phylogenetic relationship in the tree topology was statistically evaluated from 1,000 bootstrap replicates. The lynx orthopoxvirus DNA sequences obtained in this study have been submitted to the GenBank nucelotide sequence database (Table).

**Table:** Overview of 24 of 263 Eurasian lynx (*Lynx lynx)* tested for orthopoxvirus-specific DNA, Sweden*

Animal no.	GenBank accession no.	Year	Age, y†	Age class	Sex	Positive by PCR‡		
Lung	Kidney	Spleen
17§	Not sequenced	1995	ND	Subadult	F	x	x	x
23	FJ410798	1996	1	Subadult	M		x	
31§	Not sequenced	1996	1	Subadult	M	x		
38	FJ410799	1997	2	Subadult	M		x	x
49§	FJ429238	1997	ND	Adult	F			x
104	FJ429239	1997	ND	Adult	M			x
111§	FJ429240	1997	ND	Juvenile	F		x	
124	FJ429241	1998	1	Subadult	F			x
128	FJ429242	1998	1	Subadult	F			x
163§	FJ429243	1998	0	Juvenile	M	x		
165	FJ429244	1998	2	Adult	F	x		
170	FJ429245	1998	1	Subadult	F	x	x	x
197	FJ429246	1998	5	Adult	M	x		
199	FJ429247	1998	ND	Adult	M	x		
209	FJ429248	1999	ND	Subadult	M		x	x
213¶	Not sequenced	1999	1	Subadult	F			x
214	FJ429249	1999	1	Subadult	M	x	x	x
218	FJ429250	1999	1	Subadult	M	x		x
223	FJ429251	1999	4	Adult	F	x	x	
226	FJ429252	1999	0	Juvenile	F		x	
229	FJ429253	1999	4	Adult	M		x	
255	FJ429254	1999	1	Subadult	M	x	x	
270	FJ429255	1999	6	Adult	M		x	
271	FJ429256	1999	4	Adult	M		x	

*Evidenced by PCR targeting a part of the thymidine kinase gene (*tk).* Amplicons that were sequenced are underlined. ND, not determined. †Age determined by sectioning of teeth. ‡Lung, 11 (4.2%), kidney, 13 (4.9%), spleen, 11 (4.2%) of 263 samples positive. §Animals with sarcoptic mange (*Sarcoptes scabiei*); all were emaciated (other orthopoxvirus-positive animals except no. 213 were in normal condition). ¶Animal in poor condition.

### Statistical Analyses

Statistical calculations regarding differences in prevalence were performed by using NCSS 2007 Statistical Software (www.ncss.com). Statistical significance of differences was analyzed by using the 2-tailed Fisher exact test; level of significance was set at p<0.05.

## Results

In contrast to what has been reported for domestic cats and exotic felids in zoos (*11**,**12**,**33*), no macroscopic lesions in the skin or lungs could be ascribed to a cowpox virus infection. OPV PCR amplification products of expected size (≈339 bp) were detected in tissues from 24 lynx (9.1%, 95% confidence interval [CI] 5.9%–13.3%). Mean age of the 24 lynx with positive results by PCR and exact age data was 2.5 years ± 2.6 SD. Of the PCR-positive lynx, 18 were in normal body condition, 1 showed no visible signs of disease except for otodectic otoacariasis and being in poor condition, and 5 were emaciated and had sarcoptic mange (Table). Prevalence differed significantly between apparently healthy (7.7%, 95% CI 4.7%–11.7%) and diseased (29.4%, 95% CI 10.3%–55.9%; p = 0.012) lynx and between lynx in normal body condition (7.8%, 95% CI 4.7–12.1) and those that were emaciated (33.3%, 95% CI 11.8%–61.6%; p = 0.007). Most diseased and emaciated lynx had sarcoptic mange, and difference in prevalence was most significant between those with (33.3%, 95% CI 11.8%–61.6%) and without (7.7%, 95% CI 4.7%–11.7%; p = 0.007) sarcoptic mange. A significant difference was also found between areas with sparse human inhabitants (5.3%, 95% CI 1.5–12.9) and areas with higher human density (14.3%, 95% CI 8.4–22.2; p = 0.016). Yearly prevalence of animals having OPV DNA varied from 5.3% in 1997 (95% CI 1.5–12.9) to 20% in 1995 (95% CI 0.5–71.6), but differences were not significant. No significant differences were observed between sexes, age classes, or seasons.

**Figure 2:**
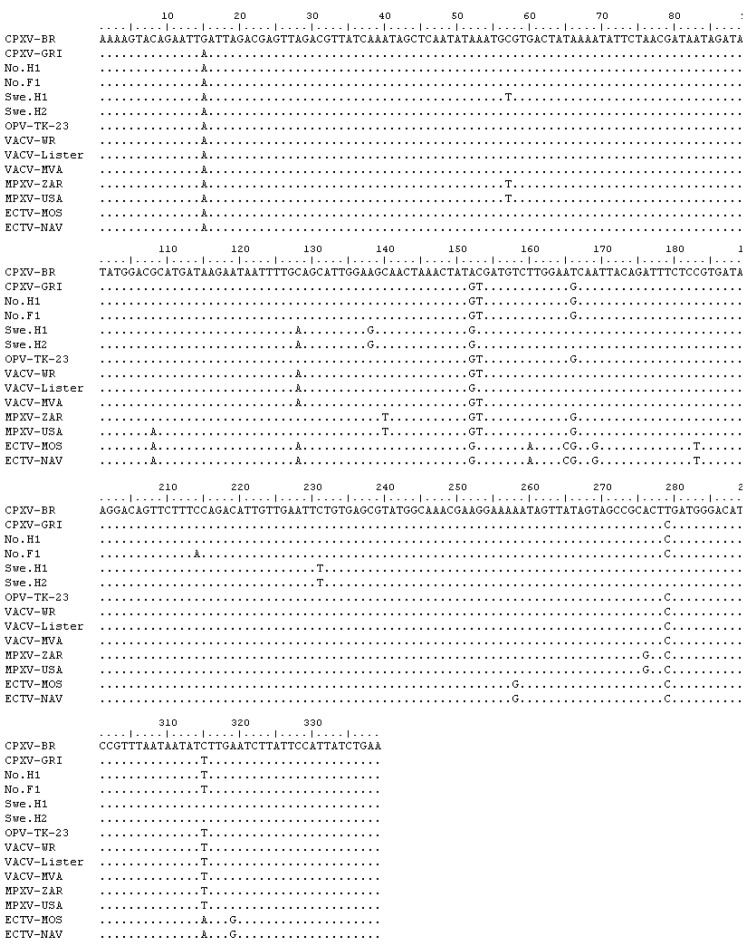
Multiple sequence alignment of the partial thymidine kinase (*tk*) gene obtained from Eurasian lynx (*Lynx lynx*) compared with the *tk* gene from other orthopoxviruses (OPVs). OPV-TK-23 represents all 21 sequences obtained from lynx tissues because they had 100% sequence homology. Swe.H1 and Swe.H2 represent 2 cowpox virus isolates from persons in Sweden. No.H1 and No.F1 represent cowpox virus isolates from a human and a felid, in Norway, respectively.

**Figure 3:**
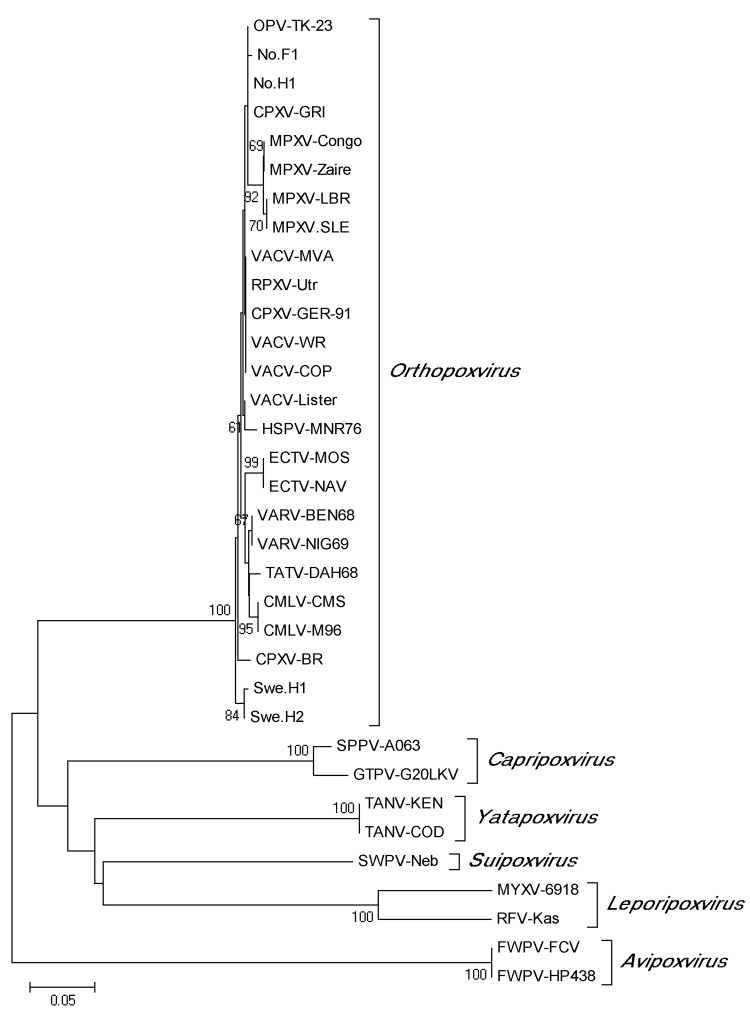
Phylogenetic tree (neighbor-joining method) generated from alignment of identical partial orthopoxvirus (OPV) thymidine kinase (*tk)* gene sequences obtained from 21 Eurasian lynx (*Lynx lynx*) from Sweden (designated OPV-TK-23) and corresponding sequences from cowpox virus isolates and other members of the genus *Orthopoxvirus* as well as other genera of the family *Poxviridae*. The corresponding *tk* gene sequences of 2 fowlpox viruses (genus *Avipoxvirus*) were used to root the tree. Only bootstrap values >60 are shown. Scale bar represents distances in substitutions per site.

Sequence data were obtained from 21 of 24 samples positive by PCR (Figure 2). The partial *tk* gene sequences were identical for all 21 samples. The obtained OPV *tk* gene sequences were also identical to that of a cowpox virus isolate from a person in Norway (No.H1) and differed by1 nt substitution from a cowpox virus isolate from a felid in Norway (No.F1) (Figure 2). In addition, the generated OPV *tk* gene sequences differed from those of CPXV-BR and of cowpox virus isolates from Sweden (Swe.H1 and Swe.H2) by multiple nucleotide substitutions (Figure 2). Phylogenetic analysis of the OPV *tk* gene sequences revealed that they clustered with the *tk* gene of cowpox virus No.H1 and cowpox virus GRI (*30*) but separated from that of cowpox virus isolates from Sweden (Swe.H1and Swe.H2) and from CPXV-BR (Figure 3).

## Discussion

On the basis of previous serologic investigations that found anti-OPV antibodies in lynx in Sweden (*25*) and our detection of OPV-specific DNA in lynx tissue samples, we conclude that the Eurasian lynx in Sweden are infected with OPV. Thus, we report finding OPV in free-ranging, wild felids.

We used the *tk* gene as a target for the PCR because it is highly conserved within species of the genus *Orthopoxvirus* (*34*), thus enabling DNA amplification of different OPVs in the sample. We cannot rule out the possibility that the OPV *tk* sequences obtained from lynx were derived from other OPVs apart from cowpox virus; but although the phylogeny is based on a partial sequence of a single gene, which may have restrictions as a predictor of phylogenetic relationships (*35*), we suggest that the *tk* OPV sequences obtained from the lynx in this study were probably derived from cowpox virus. This assumption is based on the fact that the lynx *tk* sequences were 100% identical with the partial *tk* gene of several cowpox virus isolates (Figure 2 and data not shown) and is also supported by the phylogenetic analysis, which showed that the lynx *tk* gene sequences formed a common clade with those of CPXV-GRI (strain GRI-90 isolated from a person infected by contact with a mole) (*36*) and of isolates from 1 person (No.H1) and 1 felid (No.F1) from Norway (Figure 3). Given the general genetic heterogeneity of cowpox virus isolates (*35**,**37*), it is not surprising that the clade containing the OPV *tk* sequences from lynx diverged from that of some other cowpox virus isolates, such as CPXV-BR and the 2 isolates from persons in Sweden (Swe.HI and Swe.H2; Figure 3).

Unexpectedly, the OPV sequences obtained from the lynx in Sweden were similar to the 2 isolates from a person and a felid in Norway and less similar to 2 isolates from persons in Sweden. A potential explanation is that none of these 4 clinical cases occurred near the sites in which the lynx were sampled for this study. In Sweden, the 2 human cases appeared in Skåne, the southernmost part of the country, 150–200 km from the southernmost region where the lynx were sampled. In Norway, all feline cases and all but 1 human case occurred in the southwestern part of the country, far from the common mountainous Sweden–Norway border, the region where most lynx are found. Recently, only 1 case of cowpox virus in a human has been identified in this region (Nordland County, Norway).

The presence of OPV DNA in >1 internal organs of the lynx is evidence of a virus infection and indicates that the host most likely has entered a viremic phase. No specific pathologic lesions in skin or lungs were recorded, but only a limited number of skins were submitted with the carcasses. This finding calls for increased efforts to document possible clinical signs, pathologic changes, and the effects of cowpox virus infection in lynx, taking into account the clinical signs and organ lesions documented in domestic cats and in large felid species in zoos (*11**,**12*).

Wild rodents such as bank voles, wood mice, and field voles have been suggested as the main cowpox virus reservoirs in Finland (*23*), Great Britain (*19**,**22*), and Norway (in addition to lemmings and common shrews) (*20**,**21*). Cowpox virus is considered to be endemic in these species in certain regions, circulating in several host species at the same time. In a previous examination of stomach contents of Norwegian lynx, which are part of the common lynx population inhabiting Norway and Sweden, remnants of small rodents were found in 8% of the individuals (*38*), demonstrating that rodents are part of their prey.

Domestic cats may also act as source of infection for lynx, as a potential prey species. Lynx from areas with high human density, and thus a presumably larger domestic cat population, were more often infected than lynx from less populated areas, indicating a possible relationship between domestic cats and lynx. A similar association with human presence has already been observed with regard to seropositivity to *T. gondii* in some lynx in this study (*29*). In contrast, previous studies (1999) of other pathogens in some of the lynx (n = 70) included in this study suggested that contacts between lynx and domestic cats are uncommon; seroprevalence of antibodies against feline parvovirus was low; and feline leukemia virus antigen and antibodies against feline coronavirus, calicivirus, herpesvirus, and feline immunodeficiency virus were absent (*28*). Assuming that lynx are susceptible to these feline pathogens, this difference may be explained by the fact that domestic cats are commonly vaccinated against these viral infections but not against *T. go*n*dii* and OPVs. Nevertheless, these results may suggest only limited contact between domestic cats and lynx, pointing at wild rodents and possibly shrews as the direct source of the OPV infection for lynx.

Among the 24 animals that were OPV-positive by PCR, 5 (21%) had sarcoptic mange, whereas the total prevalence of mange among the 264 animals included in this study was 6.4%; this finding indicates a possible relationship between OPV infection and infestation with *S. scabiei.* The severe epidermal lesions and breakage of the skin barrier might predispose the animals to infection by OPVs, such as cowpox virus, and such viruses might contribute to the severity of sarcoptic mange in lynx. Alternatively, altered behavior in diseased lynx could influence exposure to infection. In contrast to healthy lynx, those with mange are commonly found in human settlements, where they sometimes prey on domestic animals, including cats and dogs (*30*).

We found no seasonal differences in OPV prevalence, although one could assume that autumn, with peak populations of rodents and shrews, would be when contact rate between these animals and lynx is highest. It could also be expected that the presence of OPVs such as cowpox virus would fluctuate between years, reflecting the fluctuations of rodent populations, but we found no significant differences in prevalence between the years in this study. These findings could be the result of the low number of samples that were included from the nonhunting seasons and from some of the years represented in the study.

Cowpox virus infection is a zoonosis, capable of being transmitted from rodents to humans, often by domestic cats. Cowpox virus infection in humans is usually characterized by single lesions on the infection site (face, hands, arms), but it sometimes spreads and cause secondary lesions and complications and can be especially severe in immunocompromised persons (*6**,**16**,**23*). To our knowledge, however, only 2 verified cowpox cases in humans have been recorded from Sweden (*16**,**26*).

In conclusion, our results support the hypothesis that carnivores that prey on OPV reservoir species can be used as indicator species for the presence of such viruses in the ecosystem (*25*). This study also provides further evidence that OPV, presumably cowpox virus, is widely distributed in ecosystems in Sweden. This finding may be relevant for vaccination strategies (*39*), especially when considering the use of OPVs as vectors in genetically recombinant vaccines and their ability to undergo spontaneous genetic recombinations with virus relatives when replicating in the same cells of a host. Moreover, our findings indicate that the role of OPVs, such as cowpox virus, as potential human pathogens may increase, considering their broad distribution in ecosystems, the cessation of smallpox vaccination of humans, and an increasing number of immunocompromised persons. Thus, targeted surveillance of rodent species, and the carnivores that prey on them, is necessary for monitoring the emergence or reemergence of these viruses as potential human pathogens. Further genetic studies are needed to determine whether the detected virus in the free-ranging lynx population in Scandinavia is indeed cowpox virus.

## References

[1] Fenner F, Wittek R, Dumbell KR. The orthopoxviruses. San Diego (CA): Academic Press, Inc.; 1989. p. 432.

[2] Baxby D, Ashton DG, Jones D, Thomsett LR, Denham EM. Cowpox virus infection in unusual hosts. Vet Rec. 1979;104:175. 10.1136/vr.104.8.175-a 462730

[3] Wisser J, Pilaski J, Strauss G, Meyer H, Burck G, Truyen U, Cowpox virus infection causing stillbirth in an Asian elephant (*Elphas maximus*). Vet Rec. 2001;149:244–6. 10.1136/vr.149.8.244 11554571

[4] Martina BE, van Doornum G, Dorrestein GM, Niesters HG, Stittelaar KJ, Wolters MA, Cowpox virus transmission from rats to monkeys, the Netherlands. Emerg Infect Dis. 2006;12:1005–7. 16707063 10.3201/eid1206.051513 PMC3373046

[5] Postma BH, Diepersloot RJA, Niessen GJCM, Droog RP. Cowpox-virus–like infection associated with rat bite. Lancet. 1991;337:733–4. 10.1016/0140-6736(91)90317-I 1672197

[6] Wolfs TFW, Wagenaar JA, Niesters HGM, Osterhaus ADME. Rat-to-human transmission of cowpox infection. Emerg Infect Dis. 2002;8:1495–6. 12498670 10.3201/eid0812.020089 PMC2738512

[7] Kurth A, Wibbelt G, Gerber HP, Petschaelis A, Pauli G, Nitsche A. Rat-to-elephant-to-human transmission of cowpox virus. Emerg Infect Dis. 2008;14:670–1. 10.3201/eid1404.070817 18394293 PMC2570944

[8] Ninove L, Domart Y, Vervel C, Voinot C, Salez N, Raoult D, Cowpox virus transmission from pet rats to humans, France. Emerg Infect Dis. 2009;15:781–4. 10.3201/eid1505.090235 19402968 PMC2686997

[9] Campe H, Zimmermann P, Glos K, Bayer M, Bergemann H, Dreweck C, Cowpox virus transmission from pet rats to humans, Germany. Emerg Infect Dis. 2009;15:777–80. 10.3201/eid1505.090159 19402967 PMC2687013

[10] Enserink M. Infectious diseases. U.S. monkeypox outbreak traced to Wisconsin pet dealer. Science. 2003;300:1639. 10.1126/science.300.5626.1639a 12805511

[11] Marennikova SS, Maltseva NN, Korneeva VL, Garanina NM. Outbreak of pox disease among carnivora (Felidae) and Edentata. J Infect Dis. 1977;135:358–66. 10.1093/infdis/135.3.358 191538

[12] Baxby D, Ashton DG, Jones DM, Thomsett LR. An outbreak of cowpox in captive cheetahs: virological and epidemiological studies. J Hyg (Lond). 1982;89:365–72. 10.1017/S0022172400070935 6891393 PMC2134230

[13] Thomsett LR, Baxby D, Denham MM. Cowpox in the domestic cat. Vet Rec. 1978;108:567. 10.1136/vr.103.25.567-b 741629

[14] Schönbauer M, Schönbauer-Langle A, Kölbl S. Pockeninfektion bei einer Hauskatze. Zentralbl Veterinarmed [C]. 1982;29:434–40. 10.1111/j.1439-0450.1982.tb01245.x 6293223

[15] Bennett M, Gaskell CJ, Baxby D, Gaskell RM, Kelly DF, Naidoo J. Feline cowpox virus infection. J Small Anim Pract. 1990;31:167–73. 10.1111/j.1748-5827.1990.tb00760.x

[16] Tryland M, Myrmel H, Holtet L, Haukenes G, Traavik T. Clinical cowpox cases in Norway. Scand J Infect Dis. 1998;30:301–3. 10.1080/00365549850160972 9790141

[17] Hawranek T, Tritscher M, Muss WH, Jecel J, Nowotny N, Kolodziejek J, Feline orthopoxvirus infection transmitted from cat to human. J Am Acad Dermatol. 2003;49:513–8. 10.1067/S0190-9622(03)00762-X 12963921

[18] Haenssle HA, Kiessling J, Kempf VA, Fuchs T, Neumann C, Emmert S. Orthopoxvirus infection transmitted by a domestic cat. J Am Acad Dermatol. 2006;54:1–4. 10.1016/j.jaad.2005.09.040 16427982

[19] Crouch AC, Baxby D, McCracken CM, Gaskell RM, Bennett M. Serological evidence for the reservoir hosts of cowpox virus in British wildlife. Epidemiol Infect. 1995;115:185–91. 10.1017/S0950268800058258 7641833 PMC2271564

[20] Tryland M, Sandvik T, Mehl R, Bennett M, Traavik T, Olsvik Ø. Serosurvey for orthopoxviruses in rodents and shrews from Norway. J Wildl Dis. 1998;34:240–50. 9577770 10.7589/0090-3558-34.2.240

[21] Sandvik T, Tryland M, Hansen H, Mehl R, Moens U, Olsvik Ø, Naturally occurring orthopoxviruses: potential for recombination with vaccine vectors. J Clin Microbiol. 1998;36:2542–7. 9705389 10.1128/jcm.36.9.2542-2547.1998 PMC105159

[22] Chantrey J, Meyer H, Baxby D, Begon M, Bown KJ, Hazel SM, Cowpox: reservoir hosts and geographic range. Epidemiol Infect. 1999;122:455–60. 10.1017/S0950268899002423 10459650 PMC2809641

[23] Pelkonen PM, Tarvainen K, Hynninen A, Kallio ERK, Henttonen H, Palva A, Cowpox with severe generalized eruption, Finland. Emerg Infect Dis. 2003;9:1458–61. 14718092 10.3201/eid0911.020814 PMC3035531

[24] Henning K, Czerny CP, Meyer H, Müller T, Kramer M. A seroepidemiological survey for orthopox virus in the red fox (*Vulpes vulpes*). Vet Microbiol. 1995;43:251–9. 10.1016/0378-1135(94)00097-G 7740763

[25] Tryland M, Sandvik T, Arnemo JM, Stuve G, Olsvik Ø, Traavik T. Antibodies against orthopoxviruses in wild carnivores from Fennoscandia. J Wildl Dis. 1998;34:443–50. 9706553 10.7589/0090-3558-34.3.443

[26] Cronqvist J, Ekdal K, Kjartansdottir A, Bauer B, Klinker M. Cowpox—a cat disease in man [in Swedish]. Lakartidningen. 1991;88:2605–6. 1881217

[27] Degiorgis M-P, Lutz H, Hård af Segerstad C, Bröjer C, Bornstein S, Hoffmann-Lehmann R, Infectious diseases and other causes of death in free-ranging Eurasian lynx. Advanced Ethology. 2000;35:105.

[28] Ryser-Degiorgis MP, Hofmann-Lehmann R, Leutenegger CM, af Segerstad CH, Mörner T, Mattson R, Epizootiologic investigations in free-ranging Eurasian lynx from Sweden. J Wildl Dis. 2005;41:58–66. 15827211 10.7589/0090-3558-41.1.58

[29] Ryser-Degiorgis M-P, Jakubek E-B, Hård af Segerstad C, Bröjer C, Mörner T, Jansson DS, Serological survey of *Toxoplasma gondii* infection in free-ranging Eurasian lynx (*Lynx lynx*) from Sweden. J Wildl Dis. 2006;42:182–7. 16699164 10.7589/0090-3558-42.1.182

[30] Ryser-Degiorgis M-P, Ryser A, Bacciarini LN, Angst C, Gottstein B, Janovsky M, Notoedric and sarcoptic mange in free-ranging lynx from Switzerland. J Wildl Dis. 2002;38:228–32. 11838224 10.7589/0090-3558-38.1.228

[31] Nowicki P. Food habits and diet of the lynx (*Lynx lynx)* in Europe. J Wildl Res. 1997;2:161–6.

[32] Thompson JD, Gibson TJ, Plewniak F, Jeanmougin F, Higgins DC. The CLUSTAL_X windows interface: flexible strategies for multiple sequence alignment aided by quality analysis tools. Nucleic Acids Res. 1997;25:4876–82. 10.1093/nar/25.24.4876 9396791 PMC147148

[33] Godfrey DR, Blundell CJ, Essbauer S, Pfeffer M, Shearer DH, Rest JR, Unusual presentations of cowpox infection in cats. J Small Anim Pract. 2004;45:202–5. 10.1111/j.1748-5827.2004.tb00225.x 15116889

[34] Binns M, Mumford J, Wernery U. Analysis of the camelpox virus thymidine kinase gene. Br Vet J. 1992;148:541–6. 1467923 10.1016/0007-1935(92)90010-X

[35] Gubser C, Hue S, Kellam P, Smith GL. Poxvirus genome: a phylogenetic analysis. J Gen Virol. 2004;85:105–17. 10.1099/vir.0.19565-0 14718625

[36] Marennikova SS, Gashnikov PV, Zhukova OA, Ryabchikova EI, Streltsov VV, Ryazankina OI, Biotype and genetic characterization of the isolate of cowpox virus having caused infection in a child [in Russian]. Zh Mikrobiol. 1996;4:6–10. 9027179

[37] Hansen H, Okeke MI, Nilssen Ø, Traavik T. Comparison and phylogenetic analysis of cowpox viruses isolated from cats and humans in Fennoscandia. Arch Virol. 2009;154:1293–302. 10.1007/s00705-009-0442-5 19585075

[38] Birkeland KH, Myrberget S. The diet of the lynx (*Lynx lynx*) in Norway. Fauna Norvegica Series. 1980;A1:24–8.

[39] Damaso CRA, Esposito J, Condit RC, Moussatche N. An emergent poxvirus from humans and cattle in Rio de Janeiro State: Cantagalo virus may derive from Brazilian smallpox vaccine. Virology. 2000;277:439–49. 10.1006/viro.2000.0603 11080491

